# Mycetoma: A global medical and socio-economic dilemma

**DOI:** 10.1371/journal.pntd.0005509

**Published:** 2017-04-20

**Authors:** Ahmed H. Fahal

**Affiliations:** The Mycetoma Research Centre, WHO Collaborating Centre on Mycetoma, University of Khartoum, Khartoum, Sudan; Baylor College of Medicine, Texas Children's Hospital, UNITED STATES

Mycetoma is a common neglected tropical disease, endemic in many tropical and subtropical regions (Fig 1). Despite its distressing deformities, disability, high morbidly and negative socioeconomic impacts on patients, communities and health authorities it enjoys meagre national and international attention and recognition [1]. To date, the actual disease incidence and prevalence and infection route are not well characterised, likewise, its susceptibility, resistance and response to medical treatment. This has been reflected on the available treatment and control modalities which proved to be ineffective.

**Fig 1:**
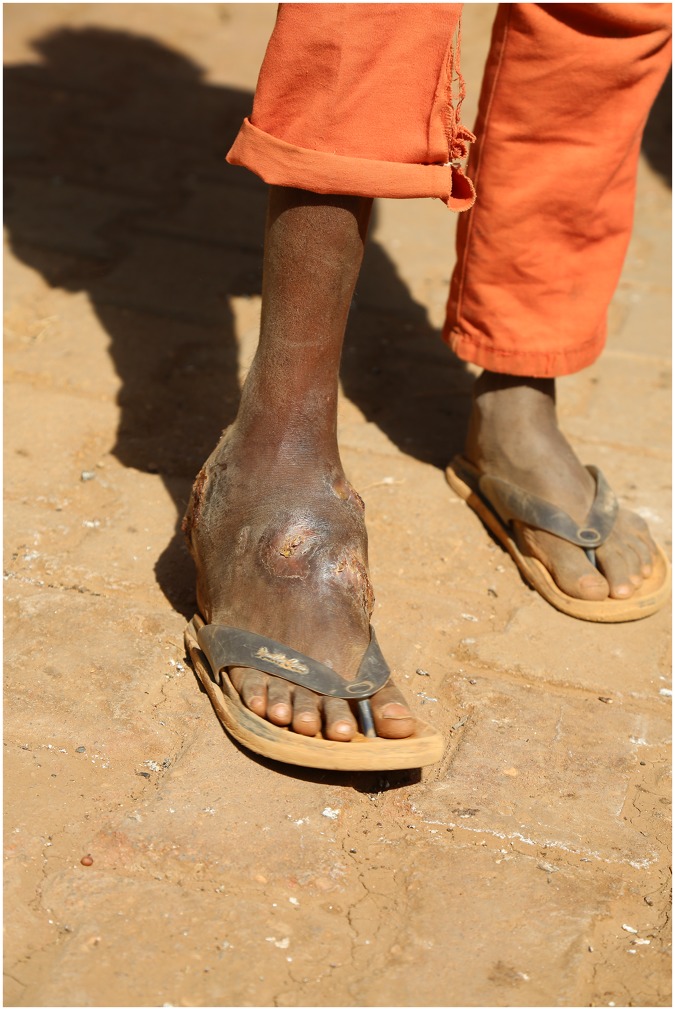
Photography showing massive neglected foot eumycetoma.

A major problem in mycetoma is that the most of the patients are of poor socio-economic and health education status and hence the late presentation, poor treatment compliance and high follow-up dropout rates. The lack of national and international attentiveness and awareness on the disease has led to a massive knowledge gap in mycetoma that had significantly and adversely affected patient care and management and proper planning for mycetoma preventative measures [2,3,4].

Mycetoma like other neglected tropical diseases needs attention. This attention is necessary for conducting basic and clinical research. The result of this research is important to improve patients’ treatment outcomes and reduce their suffering. This research provides updated information on the disease burden to patch the knowledge gap in mycetoma. Equally to raise the community awareness and to bring the treatment near to patient’s residence [3].

Accordingly, the following improvement options have been identified to address the above-mentioned dimensions of the mycetoma problems, and these are:

## Development of better disease management modalities

It is still challenging and hard to treat patients with mycetoma; in particular eumycetoma. The current treatment is still not optimal and disappointing. To cure, this disease both extensive and destructive surgery and prolonged antifungals treatment are necessary. With the current drugs, ketoconazole and itraconazole, improvement has been demonstrated, but cure rates for eumycetoma are still significantly lower than cure rates for actinomycetoma. These drugs need an extended period to affect a partial cure, they have many side effects and contraindicated in during pregnancy and lactation. The prolonged use of the available drugs proved to be not cost effective for the health authorities and patients. Surgery is curative in early cases but the fact that patients present late made its outcome to be stigmatising due to the massive mutilating surgical excisions or amputation.

A study from the Mycetoma Research Centre (MRC), Sudan, showed that of the 1242 patients with eumycetoma studied, only 321 (25.9%) were cured, 35 (2.8%) had amputations, and 54% dropped out from the outpatient follow-up. There were various reasons for the dropout but the important one was patient’s dissatisfaction [5].

Against this background, development of better treatment option which should be safe, efficacious and of short duration for mycetoma is becoming a pressing need. Since it may take long to develop a new chemical entity molecule into a drug, it is logical to make use of the available antifungal drugs and to determine their safety and efficacy. It is believed that the newer generation of azole drugs could result in a huge leap forwards in the treatment of mycetoma. Ravuconazole and Voriconazole found to be effective in-vitro testing as a good choice as a first-line treatment in patients with eumycetoma caused by *Madurella mycetomatis* [2,6].

It is becoming a trend to adopt combination therapy for treatment of many tropical neglected diseases. The advantages of this are shortening the treatment duration due to synergistic effect, reduce toxicity and cost, improve patients’ compliance and prevention drug resistance. That can be tried for mycetoma. The management of mycetoma should be holistic, addressing the different aspect of the diseases and that include, the socio-economical, medical and health aspects as well as the control and preventive measurements. The management of mycetoma can be integrated with other neglected tropical diseases [7].

## Basic research conduction to fill the knowledge gap

Still, in mycetoma, there is massive knowledge gap that is badly affecting the mycetoma patients’ management. That may be due to the patients’ perceptions of the disease and the socio-economic dislocation caused by the illness. Presently the majority of mycetoma people seek treatment too late. The explanation is multifactorial, and that includes the painless nature of the disease, the high treatment cost to the fear of amputation and lack of health education [1].

Although the mycetoma causative agents can be identified by histopathological examination of surgical biopsies, grains culture and by PCR in certain research laboratories equipped with modern techniques, there are no field-friendly diagnostic tools for early diagnosis, where the burden of disease exists. There is a need to identify purified antigens to improve the serodiagnostic tests for diagnosis and follow-up of patients on medical treatment. Furthermore, the improvement and refinement of the cultural and molecular techniques to determine the infective agent in the environment and laboratory are mandatory and essential [8,9].

Though mycetoma was reported first more than 150 years ago, still its transmission and entry route likewise the disease incidence, prevalence and mapping are not well determined globally hence detailed epidemiological studies are needed to address that. These studies are essential to design effective and objective control and preventive programmes and measurements [8].

No doubt that, the immune system plays a key role in the response of patients to medical treatment. Likewise, the resistance and susceptibility to infection. However, there are many controversies on the mycetoma patients’ immune responses to infection and treatment, and this needs considerable attention.

All issues mentioned above represent the major unreciprocated questions. Surveys and studies conduction to fill the knowledge gap is possible and necessary. However, such work needs a lot of collaboration and coordination nationally and internationally and between the affected patients, academia and health system authorities. Bridging the knowledge gaps in mycetoma will improve the quality, access and equity of health services.

## Utilisation of research results in improving patients treatment outcomes

The valid, reliable and accurate results of the basic, clinical and epidemiological research are essential to patch the massive knowledge gap in mycetoma and can be utilised to improve the patient’s management and disease control. These results will lead to the development of clinical management guidelines & protocols, field centres establishment, health providers training, and medicines procurement, to make health care to mycetoma patients universally accessible [10].

## North and south research collaboration

The North to South collaboration had resulted in the development of infrastructure and training of young researchers in developing countries and had led to the development of sound diagnostic and therapeutic agents against many neglected tropical diseases. In mycetoma, there is a reasonable international collaboration to mention but few the MRC collaboration with several international research institutes and centres, www.mycetoma.edu.sd.

The technology transfer is recognised as one of the cornerstones of development in the Third World. It is important to transfer skills, knowledge and technologies to improve the patients’ treatment and care. Technology transfer will enable local researchers, laboratory technicians, and nurses to have access to up to date instruments, equipments and technics and hence many tests and experiments can be done in developing countries, and that will reduce the cost and time. These tests will eventual be routine daily tests and not a research ones. North young scientists, researchers and post-graduate students can spend some time in the South countries for field training and gain clinical experience. Previous and ongoing experiences of collaboration between Sudan and the north in neglected diseases showed excellent results and experience. The successes in Guinea Worm, Onchocerciasis, and Trachoma projects had led to the improvement of affected communities’ health, diseases treatment and improving health equity and access to services [3,5,6,7].

## Barriers & implementation strategies

Adoption of each of the options mentioned above requires implementation of different interventions to deal with various barriers for success. These obstacles are the health system weaknesses, stakeholders’ reaction to the mentioned options, and the health providers, patients and community limitations. Furthermore, the clinical trials and basic research are costly for national bodies, the lack of national expertise in some mycetoma priority research issues and the fact that the affected mycetoma patients are poor, illiterate living in remote parts.

The overall strategies to deal with the barriers mentioned above can include the stimulation of the community leadership for mycetoma raising awareness campaigns. Nationally and internationally advocacy through reports and information sharing, stakeholders meetings and negotiations is mandatory. Improving the existing of clinical practice in mycetoma in remote areas where most the patients come from through field-based capacity building activities should be implemented [11].
